# Dependence of Work on the Pulling Speed in Mechanical Ligand Unbinding

**DOI:** 10.1021/acs.jpcb.1c01818

**Published:** 2021-07-22

**Authors:** Hong An Pham, Duc Toan Truong, Mai Suan Li

**Affiliations:** † Institute for Computational Science and Technology, QuangTrung Software City, Tan Chanh, Hiep Ward, District 12, Ho Chi Minh City 700000, Vietnam; ‡ Institute of Physics, Polish Academy Science, Al. Lotnikow 32/46, Warsaw 02-668, Poland

## Abstract

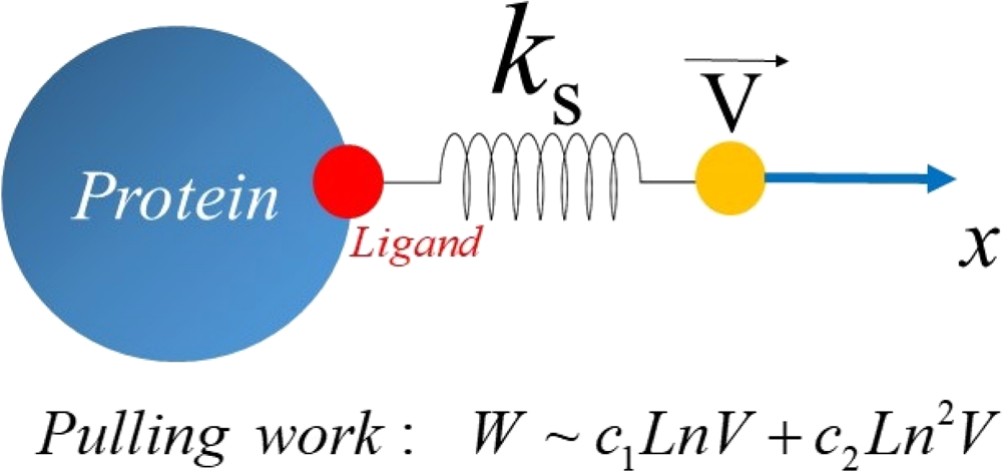

In single-molecule force spectroscopy, the rupture force *F*_max_ required for mechanical unfolding of a biomolecule or for pulling a ligand out of a binding site depends on the pulling speed *V* and, in the linear Bell–Evans regime, *F*_max_ ∼ ln(*V*). Recently, it has been found that non-equilibrium work *W* is better than *F*_max_ in describing relative ligand binding affinity, but the dependence of *W* on *V* remains unknown. In this paper, we developed an analytical theory showing that in the linear regime, *W* ∼ *c*_1_ ln(*V*) + *c*_2_ ln^2^(*V*), where *c*_1_ and *c*_2_ are constants. This quadratic dependence was also confirmed by all-atom steered molecular dynamics simulations of protein–ligand complexes. Although our theory was developed for ligand unbinding, it is also applicable to other processes, such as mechanical unfolding of proteins and other biomolecules, due to its universality.

In recent decades, single-molecule force spectroscopy (AFM, laser optical tweezers, and magnetic tweezers) has been widely used to understand biomolecular processes such as protein, RNA, and DNA unfolding, ligand unbinding, and so forth. In experiments, where the external force is ramped up at a constant speed *V*, the force–displacement/time profile contains the maximum or rupture force *F*_max_ that can be used to characterize mechanical stability of the biomolecule or binding affinity of the ligand.

The question of the dependence of the rupture force on the puling speed attracts the attention of many researchers because such a dependence can be used to extract the parameters characterizing the free energy landscape. In the linear or Bell–Evans regime,^1,2^ where *V* is small enough and it is assumed that the transition state is not shifted under an external force, Evans and Ritchie showed that^2^*F*_max_ ∼ ln(*V*), and this dependence has been confirmed by numerous experimental and simulation works (see ref (3) and references therein). To go beyond the linear regime, different scenarios of the dependence of the rupture force on the pulling speed were proposed.^4,5^

From a computational point of view, the steered molecular dynamics (SMD)^6−8^ can be used to mimic the results obtained by using single-molecule force spectroscopy. In particular, several groups have shown^9−14^ that this method is effective in predicting the relative binding affinity of small ligands to proteins based on the fact that the greater the rupture force, the higher the binding affinity. SMD can provide results as accurate as other standard methods for estimation of the binding free energy like MM-PBSA but computationally much faster^11^ as it deals with a non-equilibrium process related with fast pulling. Therefore, the use of this method is becoming more and more popular in computer-aided drug design.^9,11,13,15^

It was shown^16^ that non-equilibrium work, defined as
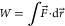
1where *r* is the ligand displacement, has a better correlation with the experiment on binding affinity than the rupture force because *W* is defined for the entire process, while *F*_max_ is calculated in only one state. This result indicates that *W* can be used as a good score for the ligand binding affinity. Despite this important fact, the dependence of the non-equilibrium work on pulling speed has not been obtained. Moreover, knowledge of this dependence should be useful for a deeper understanding of the free energy landscape of biomolecular systems, especially ligand–receptor complexes. The dependence of the average dissipated work on the displacement in the DNA hairpin pulling experiment was obtained numerically, but the analytical formula was missing.^17^ This prompted us to develop a theory by exactly solving a one-dimensional problem, which shows that at sufficiently low pulling speeds, the dependence of *W* on *V* is determined using the quadratic function of ln(*V*). We also performed all-atom simulations of protein–ligand complexes in explicit water, which confirmed our theory.

## One-Dimensional Model with Harmonic Potential

**Figure 1:**
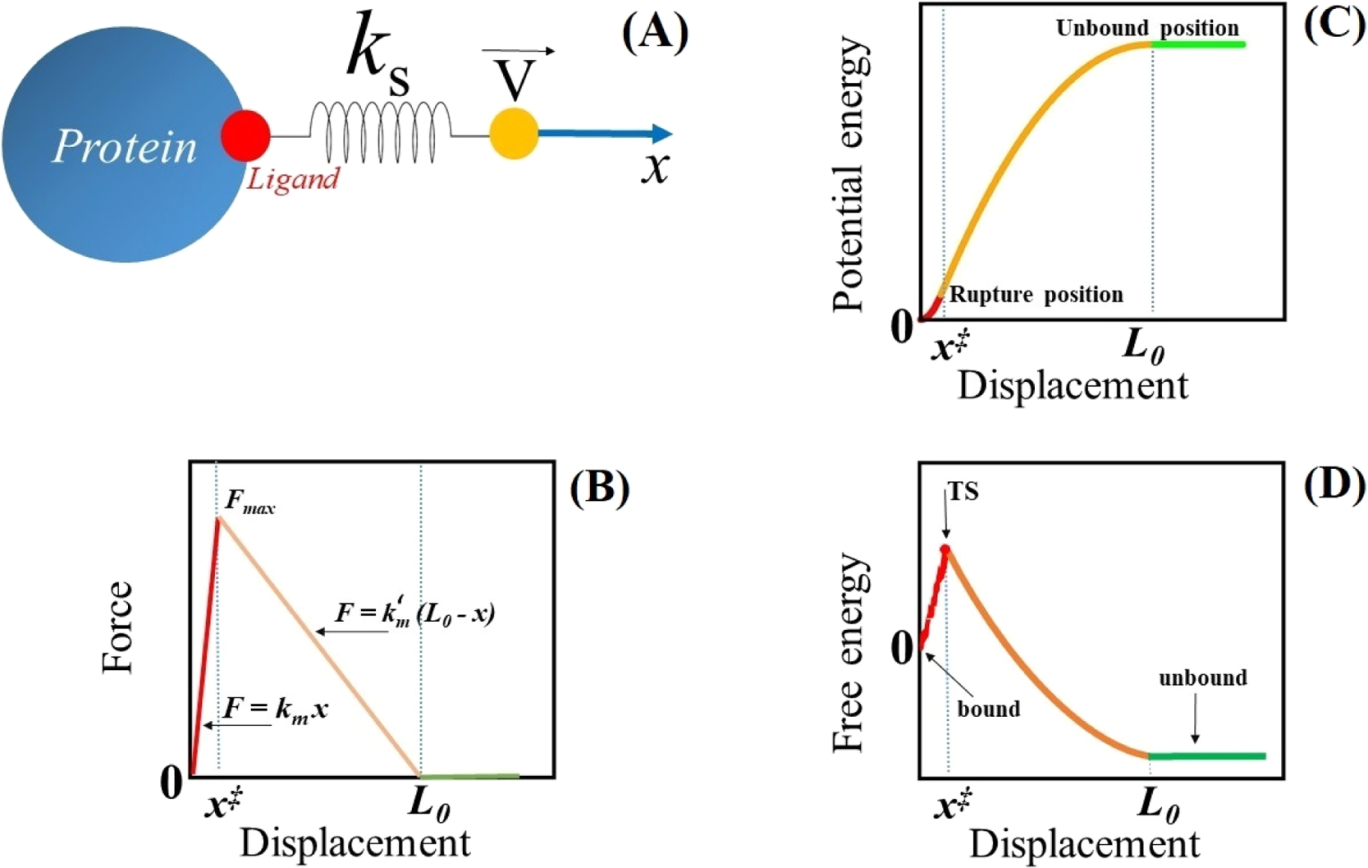
(A) Schematic description of the SMD method and single-molecule experiment. (B) Force–displacement profile: *F* = *k*_*m*_*x*, *k*′_*m*_(*L*_0_ – *x*), and 0 for *x* ≤ *x*^‡^, *x*^‡^ < *x* ≤ *L*_0_, and *x* > *L*_0_, respectively. (C) Dependence of potential energy *V*_0_(*x*) given using eq 3 on position. For simplicity, the constant in eq 3 is set to 0. (D) Conceptual graph of free energy *vs x*. The TS, which appears at *x*^‡^, separates the bound state from the unbound one.

Following Hummer and Szabo,^18^ we consider a one-dimensional motion of a ligand interacting with the receptor through potential *V*_0_(*x*). In order to mimic the single-molecule force experiment, an external force is applied to a dummy atom which is connected with the ligand using a spring with a spring constant *k*_s_ (Figure 1A). Assuming that the external force is increased at a constant speed *V*, the motion of ligand in the viscous environment is described by the following equation
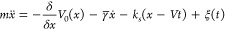
2here *x* ≡ *x*(*t*) is the time-dependent displacement, γ̅ is the Stokes friction coefficient, and ξ(*t*) is a Gaussian random force with ⟨ξ(*t*)⟩ = 0 and ⟨ξ(*t*)ξ(*t*′)⟩ = 2γ̅*k*_B_*T*δ(*t* – *t*′).

To choose an analytical expression for potential *V*_0_(*x*), we analyze a typical force–extension profile, obtained for a ligand pulled from the binding site of the protein by using all-atom SMD simulations in explicit water (Figure S1A in the Supporting Information). The ligand motion can be divided into three regimes: *x* ≤ *x*^‡^, *x*^‡^ < *x* ≤ *L*_0_, and *x* > *L*_0_, where *x*^‡^ corresponds to the position of the rupture force *F*_max_ and it is the distance from the bound sate and transition state (TS).^18,19^ Note that *x*^‡^ corresponds to the TS because, as was clearly shown in our previous work,^19^ this is the maximum of the binding free energy (see also Figure S1B) obtained by using the Jarzynski’s identity.^20,21^ For *x* ≤ *x*^‡^ and *x*^‡^ < *x* ≤ *L*_0_, the dependence of the force experienced by the ligand on the displacement can be approximated using a linear function (Figure S1A), that is, *F* = *k*_*m*_*x* and *F* = *k*′_*m*_(*L*_0_ – *x*), respectively (Figure 1B), where *k*_*m*_ and *k*′_*m*_ are spring constants. Above *L*_0_, the force and the receptor–ligand interaction disappear. Thus, *V*_0_(*x*) can be approximated using a harmonic potential as follows
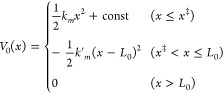
3

The constant in eq 3 can be obtained from the condition that the potential must be continuous at the rupture point *x*^‡^. In general, the spring constants *k*_*m*_ and *k*′_*m*_ differ from *k*_s_ of the cantilever.^18^*V*_0_(*x*) given by eq 3 correctly describes the fact that in the first regime (*x* ≤ *x*^‡^), the force experienced by the ligand increases and then it decreases in the second regime (*x*^‡^ < *x* ≤ *L*_0_) before vanishing at *x* > *L*_0_ (Figure 1C). In addition, the dependence of *V*_0_(*x*) on *x* (Figure 1C) is similar to that obtained from the SMD simulations (Figure S1C), which implies that our choice of potential energy is reasonable as it is supported by all-atom simulations. Based on the SMD results (Figure S1B), we can schematically describe free energy as a function of displacement (Figure 1D), which shows that ligand binding/unbinding is a barrier-crossing process. Bound and unbound states are separated by the TS, which occurs at the rupture position *x*^‡^. Our free energy profile differs, for example, from Hummer and Szabo,^18^ who were interested in the behavior of the rupture force without caring about the second regime *x*^‡^ < *x* ≤ *L*_0_. By contrast, we must take this regime into account because it contributes to the work. As in the protein folding problem, *x*^‡^ depends on the external force, but in this work, we assume it to be constant, which means that we adopt the Bell–Evans approximation.

Equation 2 with the harmonic potential (eq 3) was solved exactly, and then, using eq 1, we can obtain *W*. Because in the third region (*x* > *L*_0_), the work is zero, *W* = *W*_1_ + *W*_2_, where *W*_1_ and *W*_2_ correspond to the first and second intervals, respectively.

### Work in the First Region *x* ≤ *x*^‡^

In this region, the ligand is located in the binding site and the viscosity term in eq 2 is neglected as it is much smaller than the ligand–protein interaction term.^18^ Then, eq 2 is replaced by eq S1 in the Supporting Information with initial conditions *x*(0) = 0 and *ẋ*(0) = *v*_0_. This equation can be written as eq S2 using the Fourier transformations (eqs S3–S5) and has the exact solution for *x*(ω) (eq S6) and velocity *v*(ω) (eq S7).

The work in the first region, *W*_1_, was calculated using the definition given by eq 1 (see also eq S8 in the Supporting Information) and the expression for *F* (eq S9). After several steps (eqs S10 and S11), we obtained the work which depends on the random force (eq S12). Averaging over the random force (eqs S13–S14) and using rupture time *t*_max_ ≈ *F*_max_/*k*_s_*V*, we obtained *W*_1_ (eq S15) for one MD trajectory with a given *F*_max_.

### Work in the Second Region *x*^‡^ ≤ *x* ≤ *L*_0_

In this region, we have to keep the viscosity term and solve full eq 2. Similar to the first case, the motion equation was exactly solved (eqs S16–S19). Details of derivation of the work in the second region, *W*_2_, are described in the Supporting Information (eqs S20–S22). After averaging over the random force, we obtained the expression for *W*_2_ (eq S24).

### Dependence of the Average Total Work on the Pulling Speed

Using eqs S15 and S24, we obtain the total work
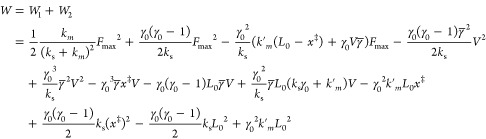
4where γ_0_ = *k*_s_/*k*_s_ – *k*′_*m*_.

In order to obtain the experimentally measurable work, we have to average *W* over the distribution of *F*_max_, <*W*> = ∫*P*(*F*_max_)*W*(*F*_max_) d*F*_max_, where in the Bell–Evans approximation, the distribution of the rupture force is given by the following expression^18^
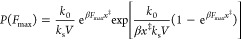
5where *k*_0_ is the intrinsic rate constant. Using the distribution given by eq 5, one can exactly calculate ⟨*F*_*max*_⟩ and ⟨*F*_max_^2^⟩^18^
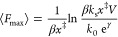
6a
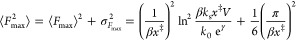
6bwith Euler constant γ = 0.577. Equation 6a describes the well-known dependence *F*_max_ ∼ ln *V*^2^, which has been widely used in interpretation of results obtained by single-molecule force spectroscopy for protein unfolding under an external force.^22,23^ This relationship is also valid for the ligand unbinding from the receptor at low loading rates.^3^

Using eq 6a to calculate the average work eq 4 ,we obtain
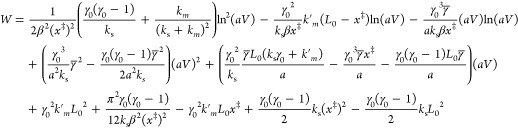
7where *aV* is the dimensionless speed with *a* = β*k*_s_*x*^‡^/*k*_0_*e*^γ^.

In the Jarzynski identity exp(−Δ*F*/*k*_B_*T*) = <exp(−*W*/*k*_B_*T*)>,^20^ we must calculate the exponential average of work over all possible trajectories connecting the states A and B. The free energy difference Δ*F* = *F*_B_ – *F*_A,_ which is obtained in equilibrium, does not depend on the pulling speed, and the exponential average of work is also independent of *V*, despite the fact that work distribution depends on the pulling speed. This can be explained by the fact that trajectories with small *W* (rare events) make the largest contribution to the exponential average. Because the process with small *W* is very close to equilibrium, <exp(−*W*/*k*_B_*T*)> does not depend on the pulling speed. In our case, we calculated the linear average of work, <*W*>, where the contribution from trajectories with large *W* (far from equilibrium) is important, and therefore, the result depends on the pulling speed.

To estimate the contribution of each term in eq 7, we rewrite the mechanical work as follows
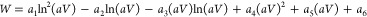
8where *a*_1_, ..., and *a*_6_, which have a unit of energy, are shown in the Supporting Information (eq S25). To estimate these coefficients, we took the typical value of the spring constant of the cantilever in the AFM experiment ∼1 *N m*^–1^,^24^ the friction coefficient γ̅ ∼ 10^–14^ kg s^–1^,^25^ and rate constant *k*_0_ ≈ 10^7^ s^–1^.^26^ The rupture position and rupture force depend on the system and pulling speed (see Figure S1B and ref (19)), but we can set *x*^‡^ ≈ 10^–10^ m and *F*_max_ ≈ 1000 pN. Then, the spring constant *k*_*m*_ ≈ *F*_max_/*x*^‡^ ≈ 1000 pN/(10^–10^ m) ≈ 10 Nm^–1^. The spring constant *k*′_*m*_ can be obtained from the condition that the first derivative of *V*_0_(*x*) with respect to *x* should be continuous at *x*^‡^, which implies that *k*_*m*_*x*^‡^ = *k*′_*m*_(*L*_0_ – *x*^‡^). Using *L*_0_ ≈ 2 nm (Figure S1A) and the values of *k*_*m*_ and *x*^‡^ given here, we obtain *k*′_*m*_ ≈ 0.5 Nm^–1^. For clarity, all parameters which will be used for estimation of *a*_1_ – *a*_6_ are shown in eq S26 in the Supporting Information. Using eq S25 and these parameters, we obtained *a*_1_ ∼ 10^–21^, *a*_2_ ∼ 10^–21^, *a*_3_ ∼ 10^–27^, *a*_4_ ∼ 10^–34^, *a*_5_ ∼ 10^–25^, and *a*_6_ ∼ 10^–18^ J (Table S1). We tested a few different sets of parameters, but the results did not change qualitatively.

It can be shown that for low pulling speeds *V* < 10^2^ nm/ns, the third, fourth, and fifth terms in eq 8 can be neglected, resulting in the following expression of the average work
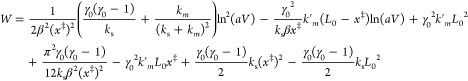
9

Because the experiment is conducted at low *V* ∼ nm/s, this dependence must be valid for its interpretation. Thus, in the Bell–Evans approximation, contrary to the rupture force case, the dependence of work on the pulling speed contains not only the ln(*V*) term but also the quadratic term ln^2^(*V*). At high pulling speeds *V* > 10^2^ nm/ns, the terms ∼*V* and *V*^2^ in eq 8 prevail over logarithmic terms, but this area is not interesting from an experimental point of view.

### SMD Simulations

To support our analytical theory (eq 9), we performed all-atom SMD simulations with explicit water for two protein–ligand complexes: the SBX small compound bound with the FKBP12 protein (PDB ID: 1FKH) and ZB6 carboxylic acid, a small compound, in complex with the AmpC beta-lactamase protein(PDB ID: 4KZ6) (Figure S2). The FKBP12 protein, consisting of 107 amino acids,^27^ is a cytosolic protein that is abundantly expressed in all tissues. It binds to FK506 and rapamycin, mediating the immunosuppressive action of drugs.^28^ Beta-lactamase,^29^ a well-known enzyme produced by bacteria,^30^ is responsible for bacterial resistance to many beta-lactam antibiotics. For clarity, the two protein–ligand complexes will hereinafter be referred as 1FKH and 4KZ6 after their PDB code.

We used the CHARMM27 force field 33^31^ and the TIP3P^32^ water model for molecular modeling. SMD simulations were carried out for a pulling speed *V* in the range 0.025–54 nm/ns, where eq 9 is applicable. Simulations below this range are beyond our computational capabilities. The number of trajectories was from 10 to 200 depending on the system and *V* (Table S2 in the Supporting Information). To mimic the AFM experiment, we chose the typical spring constant *k*_s_ = 600 kJ/(mol nm^2^) of the spring that connects the center of mass of the ligand to the dummy atom. To prevent the receptor from drifting under the influence of an external force, its Cα-atoms were restrained but the side chains were allowed to fluctuate. The details on SMD simulations are given in Supporting Information. The results obtained for *F*_max_ and *W* of two complexes with various pulling speeds are shown in Table S3.

**Figure 2:**
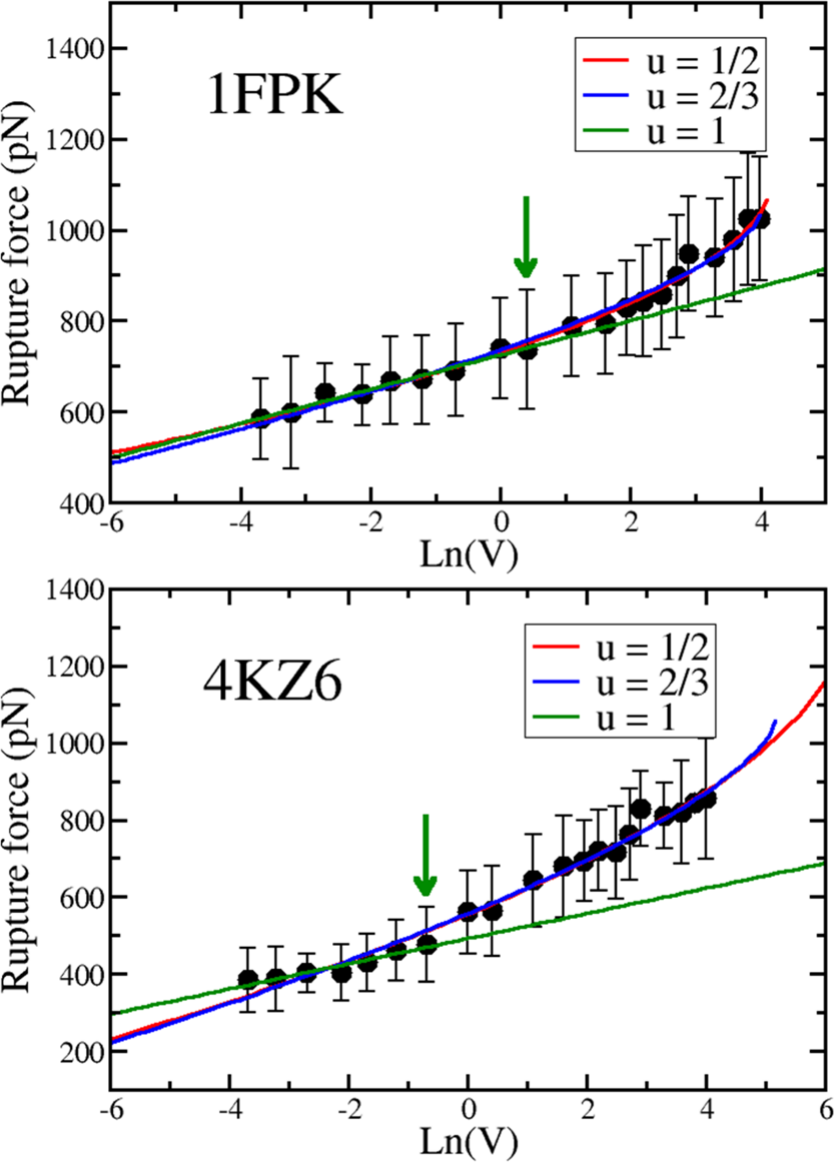
Dependence of the rupture force *F*_max_ on the logarithm of the pulling speed for complexes 1FKH (top) and 4KZ6 (bottom). The blue line is a linear fit for the first nine data points of 1FPK (*R* = 0.983) and the first seven points of 4KZ6 (*R* = 0.955). The correlation level *R* is shown in parentheses. The arrow indicates the point below which the Bell–Evans theory applies. The red and green curves are nonlinear fits using Dudko–Hummer–Szabo theory with ν = 1/2 and 2/3 for the whole data set (ν = 1 corresponds to Bell–Evans theory). For 1FPK, *R* = 0.994 and 0.992 for ν = 1/2 and 2/3, respectively, and for 4KZ6, *R* = 0.991 for both values of ν.

At low pulling speeds, the rupture force is linear with ln(*V*) (Figure 2) for both systems, but for 1FKH (the first nine points), the linear theory works over a wider range than 4KZ6 (the first seven points). The correlation level of the fit is high with *R* = 0.98 and 0.96 for 1FPK and 4KZ6, respectively. Dudko–Hummer–Szabo nonlinear theory with ν = 1/2 and 2/3^4^ is applicable to the entire region (Figure 2).

**Figure 3:**
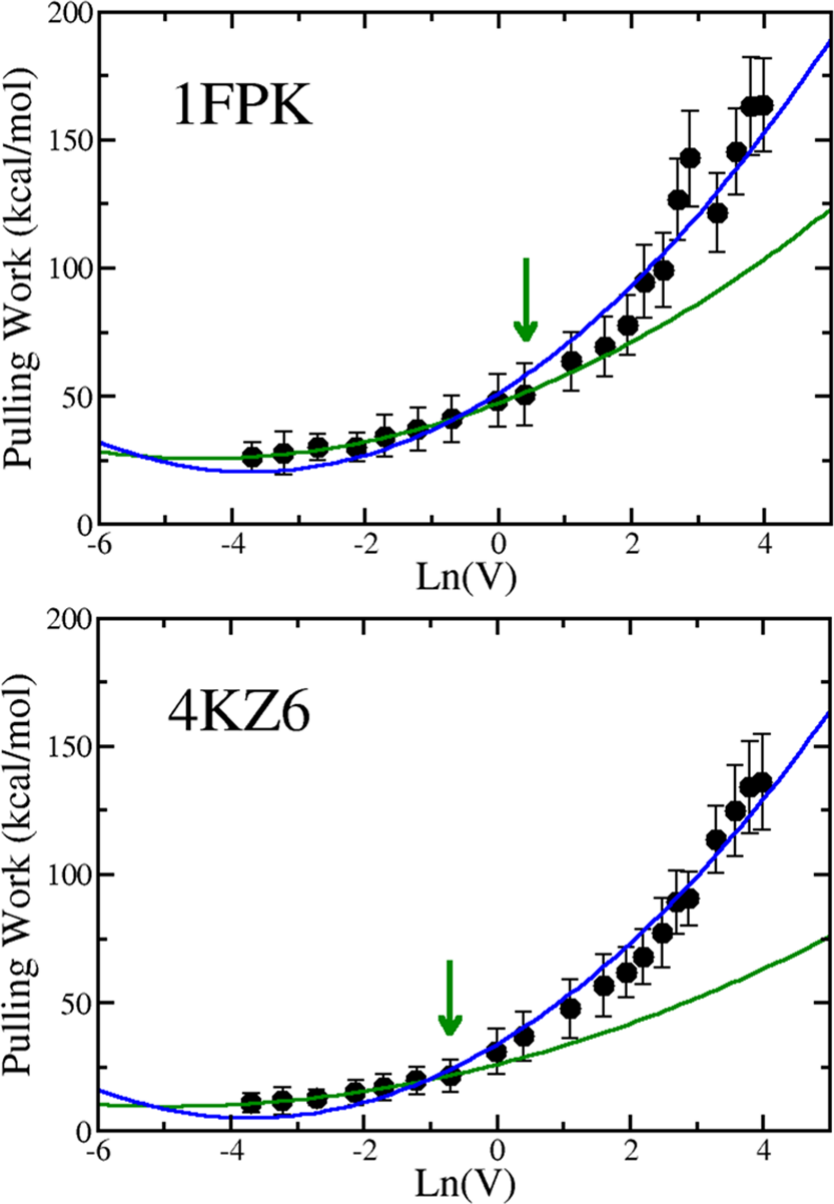
Dependence of *W* on ln(*V*) for 1FKH (top) and 4KZ6 (bottom).The arrow refers to the point below which the rupture *F*_max_ is linear with ln(*V*). The green curve is a quadratic fit for the first nine data points of 1FPK (*R* = 0.996) and the first 7 points of 4KZ6 (*R* = 0.997), which is the area where Bell–Evans theory is valid. The correlation level is indicated in parentheses. The blue curve is also the quadratic fit but for the entire data set with *R* = 0.983 and 0.988 for 4FPK and 4KZ6, respectively.

Because our theory was developed in the Bell–Evans approximation, we first applied the quadratic fit (eq 9) to the region where the rupture force linearly depends on ln(*V*), confining ourselves to the data points on the left side of the arrow in Figure 3. This fit (green curve) works perfectly for 1FPK (*R* = 0.996) and 4KZ6 (*R* = 0.997), which fully supports our theory. The blue curve in Figure 3 is a quadratic fit for the entire data set. Because the fit is good with *R* = 0.983 and 0.988 for 1FPK and 4KZ6, respectively, within error bars, our theory works for a wider range than linear. This may be due to the fact that compared with the Bell–Evans theory, we have one more fitting parameter associated with the term ln^2^(*V*). Additional protein–ligand complexes should be studied to clarify this issue.

## Conclusions

We developed a theory for the dependence of the mechanical work performed by the ligand during the escape from the receptor binding site on the pulling speed. Our exactly solvable one-dimensional model was based on the results obtained using all-atom MD simulations with explicit water. Assuming that the position of the transition state does not depend on the external force and that the receptor–ligand interaction can be described using a harmonic potential, we obtained the exact expression of *W* as a quadratic function of ln(*V*) at low enough pulling speeds. It would be interesting to confirm our theory experimentally. Although our theory was developed for ligand unbinding, it should be applied to the mechanical unfolding of proteins, RNA, and DNA and, presumably, to other more complex processes in cells.^33^ In general, the quadratic dependence (eq 9) works for the case when the force–extension profile is similar to the one shown in Figure 1B, that is, unbinding/unfolding occurs without intermediates.

Agmon and Hopfield^34^ developed a two-dimensional model of CO binding to heme proteins, in which the conceptual protein coordinate is included in addition to the CO-iron distance. This model can be used^35^ to understand recent experiments on enzyme-catalyzed reduction of disulfide bonds in proteins using the mechanical force applied to the ends of the protein.^36^ Thus, it would be interesting to extend our theory to the case where the ligand binding reaction coordinate is coupled to the protein coordinate that is responsible for the disulfide bond cleavage.^35^ This problem is challenging due to the biphasic force dependence of the bond breaking rate.

Single-molecule force spectroscopy is an effective tool for studying the breaking and formation of non-covalent protein–protein bonds, which are critical for the functions of cell adhesion complexes. It is generally believed that the external force reduces the free energy barrier to break the bond and thus shortens the bond lifetime.^2^ In contrast, Dembo *et al.*(37,38) hypothesized that force can also increase the bond lifetime by transforming the adhesive complexes into a bound state.

These two different ways of responding to external force, known as slip and grip tricks.^37,38^ By developing a phenomenological theory, Barsegov and Thirumalai showed^39^ that the dependence of the rupture force on ln(*V*) is linear in the slip regime, while it becomes more complicated (almost linear but with two different slopes) in the catch bond regime. Because our theory was developed for the case when *F*_max_ ∼ ln(*V*), it is applicable to the slip mode. Extension to the case, where catch–slip transition occurs, requires further investigation. Then, instead of one bound state in the energy landscape, one has to deal with two bound states or two pathways.^39^

In general, extension of our theory beyond the Bell–Evans approximation is of great interest. Work in this direction is in progress.

## Supporting Information Available

Typical force–displacement profiles, binding free energy and potential energy obtained by using all-atom SMD simulations at a pulling speed *V* = 5 nm/ns; derivation of the analytical expression for the pulling work; SMD simulations of 1FPK and 4KZ6 in complex with different ligands; number of trajectories used for various pulling speeds; and average rupture force and pulling work obtained by using SMD simulations for systems 1FPK and 4KZ6 (PDF)
