# Supplementary material for: Dependence of Work on the Pulling Speed in Mechanical Ligand Unbinding
Source: J Phys Chem B. 2021 Jul 22;125(30):8325–30. doi: 10.1021/acs.jpcb.1c01818 (PMC8389893; doi:10.1021/acs.jpcb.1c01818)
Supplement: Supplementary file 1 — jp1c01818_si_001.pdf [file jp1c01818_si_001.pdf]

Dependence of work on the pulling speed in mechanical ligand unbinding

Hong An Pham[1], Duc Toan Truong[1], and Mai Suan Li[2,*]

[1]Institute for Computational Science and Technology, QuangTrung Software City, Tan Chanh Hiep Ward, District 12, Ho Chi Minh City, Vietnam

[2]Institute of Physics, Polish AcadSci, Al. Lotnikow 32/46, 02-668 Warsaw, Poland.

*Correspondence: masli@ifpan.edu.pl

Supporting Information

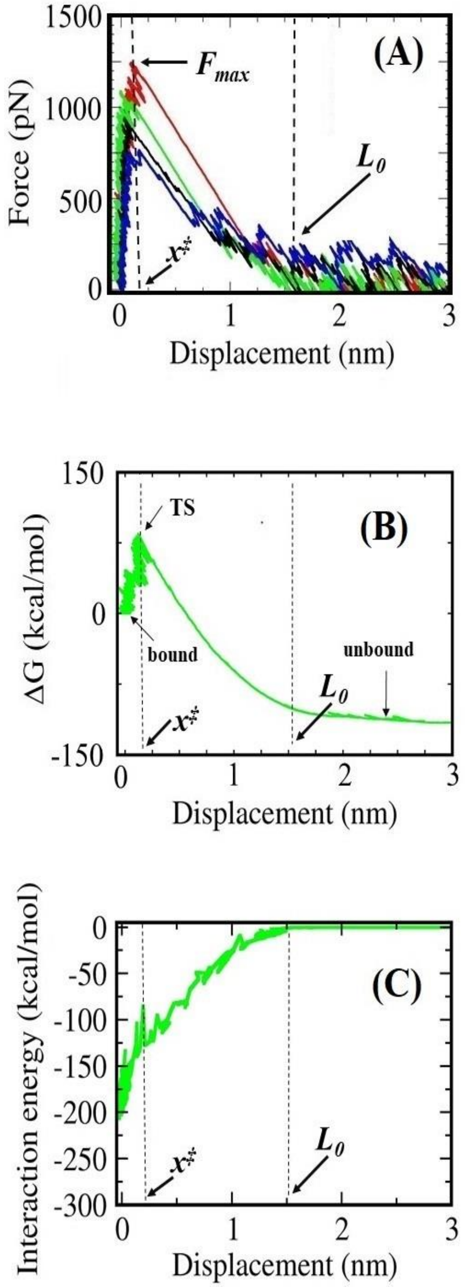

Figure S1. (A) Typical force-displacement profiles obtained by using all-atom SMD simulations for different protein-ligand complexes at the pulling speed V = 5 nm/ns. The rupture force F_{max} occurs at $\boldsymbol{x}^{\ddagger}$; for $0 \le x \le \boldsymbol{x}^{\ddagger}$ and $\boldsymbol{x}^{\ddagger} \le x \le L_0$ the force dependence on x can be approximated by a linear function. L_0 refers to the position above which the receptor-ligand interaction disappears. (B) The binding free energy ΔG as a function of x. The bound and unbound states are separated by the transition state (TS), which occurs at $\boldsymbol{x}^{\ddagger}$. (C) Dependence of the interaction energy, which includes the van der Waals and electrostatic interactions, on the ligand displacement. The result was obtained in one SMD trajectrory.

Work calculation

Region 1: $\mathbf{x} \leq \boldsymbol{x}^{\ddagger}$

According to Hummer and Szabo 2003 [1], prior to rupture, the ligand is at the binding site, and we can neglect the friction term because the velocity is low and the receptor-ligand interaction is dominant. Then we have the following motion equation:

$$m\ddot{x} = -k_m x - k_s(x - Vt) + \xi(t) \qquad \text{(S1)}$$

By using Fourier transformation we have:

$$\begin{cases} v(\omega) = -i\omega x(\omega) \\ -i\omega v(\omega) = -\omega'^2_1 x(\omega) + \frac{1}{m}\xi(\omega) - \frac{\sqrt{2\pi}}{m} i k_s V \delta'(\omega) \end{cases} \text{, (S2)}$$

where $\omega'_1 = \sqrt{\frac{k_s + k_m}{m}}$, $x(\omega)$, $v(\omega)$ and $\xi(\omega)$ are related with $x(t)$, $v(t)$ and $\xi(t)$ by the following relations

$$x(t) = \frac{1}{\sqrt{2\pi}} \int_{-\infty}^{+\infty} e^{-i\omega t} x(\omega) d\omega \quad \text{, (S3)}$$

$$v(t) = \frac{1}{\sqrt{2\pi}} \int_{-\infty}^{+\infty} e^{-i\omega t} v(\omega) d\omega \quad \text{, (S4)}$$

$$\xi(t) = \frac{1}{\sqrt{2\pi}} \int_{-\infty}^{+\infty} e^{-i\omega t} \xi(\omega) d\omega \quad \text{. (S5)}$$

From Equation S2 we obtain

$$x(\omega) = \frac{1}{m} * \frac{\xi(\omega) - \sqrt{2\pi} i k_s V \delta'(\omega)}{\omega'^2_1 - \omega^2} \quad \text{, (S6)}$$

$$v(\omega) = -i\omega x(\omega) = \frac{-i\omega}{m} * \frac{\xi(\omega) - \sqrt{2\pi} i k_s V \delta'(\omega)}{\omega'^2_1 - \omega^2} \text{, (S7)}$$

Then we calculate the work in region 1:

$$W_1 = \int F dx = \int F \frac{dx}{dt} dt = \int F v dt \,. \quad \text{(S8)}$$

Using

$$F = k_s V t - k_s x \qquad \text{(S9)}$$

and Equations S3, S4, S6, S7, and S9 we have

$$W_1 = \int \left(k_s V t - \frac{k_s}{\sqrt{2\pi}} \int_{-\infty}^{+\infty} e^{-i\omega t} x(\omega) d\omega \right) * \frac{1}{\sqrt{2\pi}} \int_{-\infty}^{+\infty} e^{-i\omega t} v(\omega) d\omega * dt \quad \text{(S10)}$$

Substituting $x(\omega)$ and $v(\omega)$ from Equations S6 and S7 into the last equation we obtain:

$$W_1 = \int \left(k_s V t - \frac{k_s}{\sqrt{2\pi}} \int_{-\infty}^{+\infty} e^{-i\omega t} \frac{1}{m} * \frac{\xi(\omega) - \sqrt{2\pi} i k_s V \delta'(\omega)}{\omega'^2_1 - \omega^2} d\omega \right) * \left(\frac{1}{\sqrt{2\pi}} \int_{-\infty}^{+\infty} e^{-i\omega t} \frac{-i\omega}{m} * \frac{\xi(\omega) - \sqrt{2\pi} i k_s V \delta'(\omega)}{\omega'^2_1 - \omega^2} d\omega \right) dt \text{ (S11)}$$

Using $f(x)\delta'(x) = -f'(x)\delta(x)$,

$$W_1 = \int_0^{t_{max}} \left(k_s V t + \frac{k_s k_s V}{m}\left(-\frac{t}{\omega'^2_1}\right) - \frac{k_s}{\sqrt{2\pi}}\int_{-\infty}^{+\infty} e^{-i\omega t}\frac{1}{m} * \frac{\xi(\omega)}{\omega'^2_1-\omega^2}d\omega\right) * \left(\frac{1}{\sqrt{2\pi}}\int_{-\infty}^{+\infty} e^{-i\omega t}\frac{-i\omega}{m} * \frac{\xi(\omega)}{\omega'^2_1-\omega^2}d\omega + \frac{1}{m}\frac{k_s V}{\omega'^2_1}\right)dt \quad \text{(S12)}$$

Then taking average over the random force we have:

$$W_1 = \int_0^{t_{max}} \left\{\frac{1}{m}\frac{k_s V}{\omega'^2_1}k_s V t - \frac{k_s}{m^2} * \frac{k_s V}{\omega'^4_1}k_s V t + \frac{k_s}{2\pi}\frac{i}{m^2}\int_{-\infty}^{+\infty}\omega' * e^{-i(\omega+\omega')t} * \frac{\langle\xi(\omega)\xi(\omega\prime)\rangle}{\left(\omega'^2_1-\omega^2\right)\left(\omega'^2_1-\omega'^2\right)}d\omega d\omega'\right\}dt \quad \text{(S13)}$$

From the equation $\langle\xi(t)\xi(t')\rangle = 2\bar{\gamma}k_B T\delta(t-t')$, we have

$$\langle\xi(\omega)\xi(\omega')\rangle = 2\bar{\gamma}k_B T\delta(\omega+\omega') \quad \text{(S14)}$$

Using this equation and rupture time $t_{max} \approx \frac{F_{max}}{k_s V}$ we obtain

$$W_1 = \frac{1}{2}\frac{k_m}{(k_s+k_m)^2}{F_{max}}^2 . \quad \text{(S15)}$$

Region 2: $x^{\ddagger} < x \le L_0$

After rupture, the viscosity term should be taken into account, because the ligand speed becomes greater than in the first region. Then we have:

$$m\ddot{x} = k'_m(x-L_0) - k_s(x-vt) - \bar{\gamma}\dot{x} + \xi(t) \qquad \text{(S16)}$$

By using Fourier transformation obtain:

$$\begin{cases} v(\omega) = -i\omega x(\omega) \\ -i\omega v(\omega) = -\omega_0^2 x(\omega) - \frac{\bar{\gamma}}{m}v(\omega) + \frac{1}{m}\xi(\omega) - \frac{\sqrt{2\pi}}{m}k'_m L_0\delta(\omega) - \frac{\sqrt{2\pi}}{m}ik_s V\delta'(\omega) \end{cases} \quad \text{(S17)}$$

where $\omega_0 = \sqrt{\frac{k_s-k'_m}{m}}$. Then from the last equation we have

$$x(\omega) = \frac{1}{m} * \frac{\xi(\omega)-\sqrt{2\pi}k'_m L_0\delta(\omega)-\sqrt{2\pi}ik_s V\delta'(\omega)}{\omega_0^2-\omega^2-\frac{\bar{\gamma}}{m}i\omega} , \qquad \text{(S18)}$$

$$v(\omega) = -i\omega x(\omega) = \frac{-i\omega}{m} * \frac{\xi(\omega)-\sqrt{2\pi}k'_m L_0\delta(\omega)-\sqrt{2\pi}ik_s V\delta'(\omega)}{\omega_0^2-\omega^2-\frac{\bar{\gamma}}{m}i\omega} \quad \text{(S19)}$$

Similar to the region 1 case, the work was calculated by using Equations S8-S10:

$$W_2 = \int_{x^{\ddagger}}^{L_0} F dx = \int_{x^{\ddagger}}^{L_0} F\frac{dx}{dt}dt = \int_{t_{x^{\ddagger}}}^{t_{L_0}} F v dt = \int_{t_{x^{\ddagger}}}^{t_{L_0}} \left(k_s V t - \frac{k_s}{\sqrt{2\pi}}\int_{-\infty}^{+\infty} e^{-i\omega t}x(\omega)d\omega\right) * \frac{1}{\sqrt{2\pi}}\int_{-\infty}^{+\infty} e^{-i\omega t}v(\omega)d\omega * dt \quad \text{(S20)}$$

Substituting $x(\omega)$ and $v(\omega)$ from Equations S18 and S19 into this equation we arrive at

$$W_2 = \int_{t_{max}}^{t_{L_0}} \left(k_s V t - \frac{k_s}{\sqrt{2\pi}}\int_{-\infty}^{+\infty} e^{-i\omega t}\frac{1}{m} * \frac{\xi(\omega)-\sqrt{2\pi}k'_m L_0\delta(\omega)-\sqrt{2\pi}ik_s V\delta'(\omega)}{\omega_0^2-\omega^2-\frac{\bar{\gamma}}{m}i\omega}d\omega\right) * \left(\frac{1}{\sqrt{2\pi}}\int_{-\infty}^{+\infty} e^{-i\omega t}\frac{-i\omega}{m} * \frac{\xi(\omega)-\sqrt{2\pi}k'_m L_0\delta(\omega)-\sqrt{2\pi}ik_s V\delta'(\omega)}{\omega_0^2-\omega^2-\frac{\bar{\gamma}}{m}i\omega}d\omega\right)dt \quad \text{(S21)}$$

$$W_2 = \int_{t_{max}}^{t_{L_0}} \left(k_s V t + \frac{k_s}{m} * \frac{k'_m L_0}{\omega_0^2} + \frac{k_s k_s V}{m} \left(-\frac{t}{\omega_0^2} + \frac{\frac{\bar{\gamma}}{m}}{\omega_0^4} \right) - \frac{k_s}{\sqrt{2\pi}} \int_{-\infty}^{+\infty} e^{-i\omega t} \frac{1}{m} * \frac{\xi(\omega)}{\omega_0^2 - \omega^2 - \frac{\bar{\gamma}}{m} i\omega} d\omega \right) * \left(\frac{1}{\sqrt{2\pi}} \int_{-\infty}^{+\infty} e^{-i\omega t} \frac{-i\omega}{m} * \frac{\xi(\omega)}{\omega_0^2 - \omega^2 - \frac{\bar{\gamma}}{m} i\omega} d\omega + \frac{1}{m} \frac{k_s V}{\omega_0^2} \right) dt \,. \quad \text{(S22)}$$

Averaging over the random force, we obtain

$$W_2 = \int_{t_{max}}^{t_{L_0}} \left\{ \frac{1}{m} \frac{k_s V}{\omega_0^2} k_s V t - \frac{k_s}{m^2} * \frac{k_s V}{\omega_0^4} k_s V t + \frac{k_s}{m^2} * \frac{k'_m L_0}{\omega_0^4} k_s V + \frac{k_s}{m^3} * \frac{k_s V}{\omega_0^6} k_s V \bar{\gamma} + \frac{k_s}{2\pi} \frac{i}{m^2} \int_{-\infty}^{+\infty} \omega' * e^{-i(\omega+\omega')t} * \frac{\langle \xi(\omega) \xi(\omega') \rangle}{\left(\omega_0^2 - \omega^2 - \frac{\bar{\gamma}}{m} i\omega \right) \left(\omega_0^2 - \omega'^2 - \frac{\bar{\gamma}}{m} i\omega' \right)} d\omega d\omega' \right\} dt \quad \text{(S23)}$$

Using $\langle \xi(\omega)\xi(\omega') \rangle = 2\bar{\gamma} k_B T \delta(\omega + \omega')$, $t_{max} \approx \frac{F_{max}}{k_s V}$, $t_{L_0} = \frac{F_{L_0} + k_s L_0}{k_s V}$ and $F_{L_0} = \bar{\gamma} V$, then we have

$$W_2 = \left\{ \frac{\gamma_0(\gamma_0 - 1)}{2k_s} F_{max}{}^2 - \frac{\gamma_0{}^2}{k_s} (k'_m(L_0 - x^{\ddagger}) + \gamma_0 V \bar{\gamma}) F_{max} - \frac{\gamma_0(\gamma_0 - 1)\bar{\gamma}^2}{2k_s} V^2 + \frac{\gamma_0{}^3}{k_s} \bar{\gamma}^2 V^2 - \gamma_0{}^3 \bar{\gamma} x^{\ddagger} V - \gamma_0(\gamma_0 - 1) L_0 \bar{\gamma} V + \frac{\gamma_0{}^2}{k_s} \bar{\gamma} L_0 (k_s \gamma_0 + k'_m) V - \gamma_0{}^2 k'_m L_0 x^{\ddagger} + \frac{\gamma_0(\gamma_0 - 1)}{2} k_s x^{\ddagger 2} - \frac{\gamma_0(\gamma_0 - 1)}{2} k_s L_0{}^2 + \gamma_0{}^2 k'_m L_0{}^2 \right\} \quad \text{(S24)}$$

where $\gamma_0 = \frac{k_s}{(k_s - k'_m)}$.

Coefficients $a_1 - a_6$ in Eq. 8 in the main text

$$\begin{cases} a_1 = \frac{1}{2\beta^2 x^{\ddagger 2}} \left(\frac{\gamma_0(\gamma_0 - 1)}{k_s} + \frac{k_m}{(k_s + k_m)^2} \right) \\ a_2 = \frac{\gamma_0{}^2}{k_s \beta x^{\ddagger}} k'_m (L_0 - x^{\ddagger}) \\ a_3 = \frac{\gamma_0{}^3 \bar{\gamma}}{a k_s \beta x^{\ddagger}} \\ a_4 = \frac{\gamma_0{}^3}{a^2 k_s} \bar{\gamma}^2 - \frac{\gamma_0(\gamma_0 - 1)\bar{\gamma}^2}{2a^2 k_s} \\ a_5 = \frac{\gamma_0{}^2}{k_s} \frac{\bar{\gamma} L_0 (k_s \gamma_0 + k'_m)}{a} - \frac{\gamma_0{}^3 \bar{\gamma} x^{\ddagger}}{a} - \frac{\gamma_0(\gamma_0 - 1) L_0 \bar{\gamma}}{a} \\ a_6 = \gamma_0{}^2 k'_m L_0{}^2 + \frac{\pi^2 \gamma_0(\gamma_0 - 1)}{12 k_s \beta^2 x^{\ddagger 2}} - \gamma_0{}^2 k'_m L_0 x^{\ddagger} + \frac{\gamma_0(\gamma_0 - 1)}{2} k_s x^{\ddagger 2} - \frac{\gamma_0(\gamma_0 - 1)}{2} k_s L_0{}^2 \end{cases} \quad \text{(S25)}$$

$$
\begin{cases}
\bar{\gamma} \approx 10^{-14}\ kg.s^{-1} \\
k_s = 1\ N.m^{-1} \\
k_m \approx 10\ N.m^{-1} \\
k'_m \approx 0.5\ N.m^{-1} \\
L_0 \approx 2.10^{-9}\ m \\
x^{\ddagger} \approx 10^{-10}\ m \\
k_0 \approx\ 10^{7}\ s^{-1} \\
\beta \approx 2.42 * 10^{20}\ kg^{-1}m^{-2}s^{2} \\
\gamma = 0.577 \\
\gamma_0 = \frac{k_s}{(k_s - k'_m)} \approx 2 \\
a = \frac{\beta k_s x^{\ddagger}}{k_0 e^{\gamma}} \approx 1.4 * 10^{3}\ s.m^{-1}
\end{cases}
\quad \text{(S26)}
$$

Substituting these parameters into Eq. (S25) we obtained the coefficients $a_1 - a_2$ in Table S1.

Coefficients	a_1	a_2	a_3	a_4	a_5	a_6
Estimated value	$1.8 * 10^{-21}$	$4.2 * 10^{-21}$	$2 * 10^{-27}$	$3.6 * 10^{-34}$	$1.1 * 10^{-25}$	$3.6 * 10^{-18}$

Table S1: Typical values of coefficients $a_1 - a_2$ measured in J.

Molecular simulations

Choice of protein-ligand complexes

To support our theory we performed molecular simulations for two receptor-ligand complexes. The first complex, which has the PDB ID 1FKH [2] (Figure S2), contains a small SBX compound located at the binding site of the FKBP12 protein. Another complex (PDB ID: 4KZ6[3]) consists the AmpC beta-lactamase protein and the ZB6 carboxylic acid, a small compound (Figure S2). The FKBP12 protein [4] has 107 amino acids; it is a 12 kDa cytosolic protein that is abundantly expressed in all tissues. It binds to FK506 and rapamycin, which mediates the immunosuppressive effect of drugs [5]. Binding to FKBP12 allows drugs to subsequently interact with mechanistic targets of their action in immunosuppression [6].

Beta-lactamase[7], an enzyme produced by bacteria [8], is responsible for the resistance of bacteria to many beta-lactam antibiotics like penicillins, cephalosporins and carbapenems. A typical AmpC protein consists of 716 amino acids with approximately 6310 atoms and a mass of 79.88 kDa. Suppression of AmpC activity can prevent bacterial resistance [9, 10].

Preparation of initial conformations for molecular dynamics simulations

From the PDB protein data bank, the ligand – protein conformation was downloaded and then the pdb format file was loaded by Pymol [11] software version 2.0 and separately saved into two files for a protein and a ligand. For the ligand conformation, an auto geometry optimization and an atomH adding were performed by Avogadro [12] package, and the resulting file must be saved in MOL2 format. Missing hydrogen atoms were added to the protein conformation by using the GROMACS [13] package version 2016.5 with the CHARMM27 [14] force field 33 and the TIP3P [15] water model.

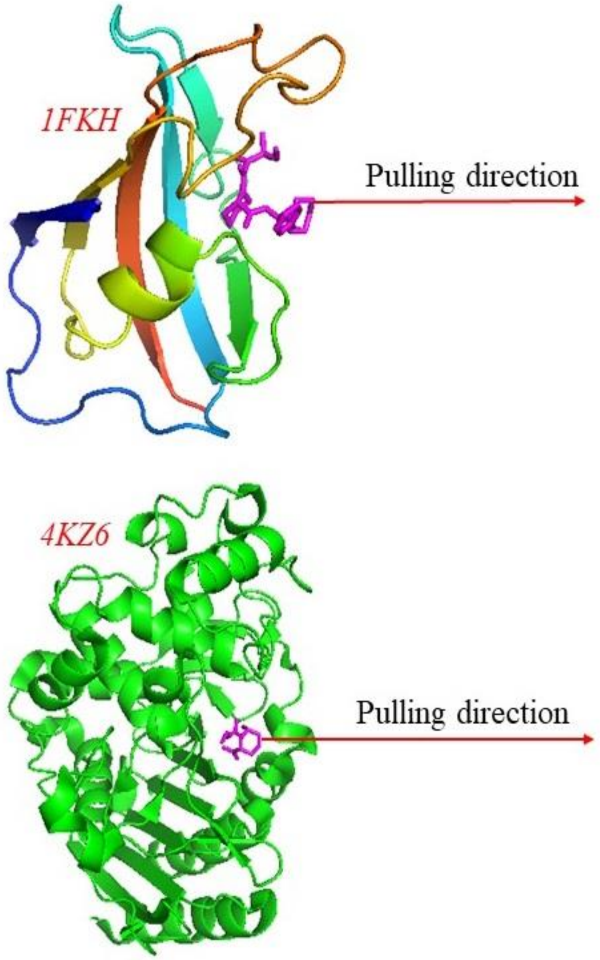

Figure S2. Top: Crystal structure (PDB ID: 1FKH [2]) of a SBX small compound bound to the FKBP12 protein. Bottom: Crystal structure (PDB ID: 4KZ6 [3]) of a small carboxylic acid compound ZB6 complexed with the beta-lactamase protein AmpC. The arrow refers to the pulling direction.

Molecular dynamics simulations

Molecular dynamics (MD) simulation was performed using the Gromars package version 2016.5. The solvated box was set large enough to ensure that the protein does not interact with itself. The SwissParam [16] server (http://www.swissparam.ch) was used to create the ligand conformation topology file. After optimization, the system, including the receptor-ligand complex, solvent and counter ions, was equilibrated through three steps: energy minimization, 500 ps simulation in NVT and 500ps simulation in NPT ensemble. The temperature and pressure in our simulations were 300 K and 1atm, respectively. Full systems with water contain approximately 30000 and 20000 atoms for 4KZ6 and 1FPK, respectively.

Steered molecular dynamic simulations

In the steered molecular dynamics (SMD) simulations an external force $F=k_s(\Delta x-vt)$ was applied to a dummy atom that connects to the ligand through a spring with the spring constant k_s, v is the pulling speed and Δx is the displacement of the pulled atom of the ligand from its initial position. To simulate the AFM experiment [17], we chose the cantilever stiffness $k_s = 600\ \frac{kJ}{mol.nm^2}$. The pulling direction of the ligand from the binding site was determined using the minimal steric hindrance method [18] (Figure S2). All Cα atoms of the receptor were restrained to avoid its drift under the influence of an external force. To obtain reliable results we carried out many SMD trajectories and their number depends on the system and the pulling speed (Table S2). With a slow pull, the errors are smaller and fewer runs are required.

From the force-displacement/time profile we collected the rupture force for the calculation of $\langle F_{max} \rangle$. The pulling work for a given trajectory was calculated using Equation (1) in the main text and the trapezoid rule:

$$W = \int F(x)dx = \frac{1}{2}\sum_{i=1}^{n-1}(f_{i+1} + f_i)(x_{i+1} - x_i).$$

Results obtained for the average rupture force, rupture time and pulling works are shown in Table S3.

	Number of trajectories	
Pulling speed (m/s)	4KZ6	1FKH
0.025	10	20
0.04	10	20
0.067	20	20
0.12	20	40
0.183	20	40
0.3	20	50
0.5	50	50
1	50	50
1.5	50	100
3	50	100
5	100	100
7	200	200
9	200	200
12	200	200
15	200	200
18	200	200
27	200	200
36	200	200
45	200	200
54	200	200

Table S2: Number of trajectories used for various pulling speeds.

	1FKH		*4KZ6*	
Pulling speed (m/s)	$\langle F_{\max} \rangle$ (pN)	$\langle W_{pull} \rangle$ (kcal/mol)	$\langle F_{\max} \rangle$ (pN)	$\langle W_{pull} \rangle$ (kcal/mol)
0.025	584.1 ± 89	26 ± 5.7	384.1 ± 84.2	10.8 ± 3.7
0.04	598.2 ± 123.8	27.6 ± 8.2	387.6 ± 84	11.6 ± 5.4
0.067	641.1 ± 64.6	29.9 ± 5.3	402.1 ± 50.5	12.8 ± 3.1
0.12	637.7 ± 66.6	30 ± 5.6	403.8 ± 74	14.8 ± 4.7
0.183	668.3 ± 96.6	34.3 ± 8.1	431.3 ± 74.3	16.7 ± 5.2
0.3	671.3 ± 97.5	37.1 ± 8.4	462.7 ± 78.5	19.5 ± 5.2
0.5	691.5 ± 100.9	40.9 ± 9.2	477.7 ± 97.5	21.4 ± 6.4
1	738.5 ± 110.7	48.3 ± 10.3	560.4 ± 107.6	31 ± 8.8
1.5	736.1 ± 130.7	50.6 ± 12.1	563.7 ± 116	36.8 ± 9.7
3	788.9 ± 110.1	63.4 ± 11.6	642.2 ± 119.5	47.7 ± 11.4
5	793.9 ± 110.9	69 ± 11.7	679.4 ± 132.6	56.6 ± 12.3
7	828.1 ± 104	77.5 ± 11.6	692.8 ± 104.8	61.8 ± 9.7
9	842.9 ± 121.4	94.5 ± 14.3	720.3 ± 104.2	67.6 ± 10.8
12	857.8 ± 120.2	99.1 ± 14.5	715.4 ± 121.1	77.1 ± 13.7
15	897.1 ± 133.8	126.5 ± 16	762.7 ± 117.9	89 ± 12.4
18	947 ± 125.3	161.3 ± 18.7	829.3 ± 97.4	90.4 ± 10.4
27	939 ± 129.5	121.6 ± 15.5	810.6 ± 85.5	113.4 ± 13.1
36	978 ± 135.5	145.2 ± 16.7	819.4 ± 134	124.7 ± 17.8
45	1023.4 ± 144.6	162.9 ± 19.1	844.5 ± 3.4	133.8 ± 17.9
54	1024.1 ± 136.7	163.4 ± 18.1	854.7 ± 157.3	135.9 ± 18.5

Table S3: Average rupture force and pulling work obtained by using SMD simulations for two complexes. Error bars are standard deviations.

References

[1] G. Hummer, A. Szabo, Kinetics from nonequilibrium single-molecule pulling experiments, Biophysical journal 85 (2003) 5-15.
[2] D.A. Holt, J.I. Luengo, D.S. Yamashita, H.J. Oh, A.L. Konialian, H.K. Yen, L.W. Rozamus, M. Brandt, M.J. Bossard, M.A. Levy, Design, synthesis, and kinetic evaluation of high-affinity FKBP ligands and the X-ray crystal structures of their complexes with FKBP12, Journal of the American Chemical Society 115 (2002) 9925-9938.
[3] S. Barelier, O. Eidam, I. Fish, J. Hollander, F. Figaroa, R. Nachane, J.J. Irwin, B.K. Shoichet, G. Siegal, Increasing chemical space coverage by combining empirical and computational fragment screens, ACS chemical biology 9 (2014) 1528-1535.
[4] T. Clackson, W. Yang, L.W. Rozamus, M. Hatada, J.F. Amara, C.T. Rollins, L.F. Stevenson, S.R. Magari, S.A. Wood, N.L. Courage, Redesigning an FKBP–ligand interface to generate chemical dimerizers with novel specificity, Proceedings of the National Academy of Sciences 95 (1998) 10437-10442.
[5] M.T. Ivery, Immunophilins: switched on protein binding domains?, Medicinal research reviews 20 (2000) 452-484.
[6] G. Hamilton, J. Steiner, Immunophilins: beyond immunosuppression, Journal of medicinal chemistry 41 (1998) 5119-5143.
[7] F.K. Majiduddin, I.C. Materon, T.G. Palzkill, Molecular analysis of beta-lactamase structure and function, International journal of medical microbiology 292 (2002) 127.
[8] H.E. Noor-ul-Ain Jameel, A. Zafar, H. Amin, Multidrug resistant AmpC β-lactamase producing Escherichia coli isolated from a paediatric hospital, Pakistan journal of medical sciences 30 (2014) 181.
[9] J. Yatsuyanagi, S. Saito, T. Konno, S. Harata, N. Suzuki, J. Kato, K.-i. Amano, Nosocomial outbreak of ceftazidime-resistant Serratia marcescens strains that produce a chromosomal AmpC variant with N235K substitution, Japanese journal of infectious diseases 59 (2006) 153.
[10] T. Vanwynsberghe, K. Verhamme, M. Raymaekers, R. Cartuyvels, K.V. Vaerenbergh, A. Boel, H.d. Beenhouwer, A large hospital outbreak of Klebsiella pneumoniae (DHA-1 and SHV-11 positive): Importance of detection and treatment of AmpC β-lactamases, The Open Infectious Diseases Journal 3 (2009).
[11] W.L. DeLano, Pymol: An open-source molecular graphics tool, CCP4 Newsletter on protein crystallography 40 (2002) 82-92.
[12] M.D. Hanwell, D.E. Curtis, D.C. Lonie, T. Vandermeersch, E. Zurek, G.R. Hutchison, Avogadro: an advanced semantic chemical editor, visualization, and analysis platform, Journal of cheminformatics 4 (2012) 17.
[13] E. Lindahl, B. Hess, D. Van Der Spoel, GROMACS 3.0: a package for molecular simulation and trajectory analysis, Molecular modeling annual 7 (2001) 306-317.
[14] A.D. MacKerell Jr, N. Banavali, N. Foloppe, Development and current status of the CHARMM force field for nucleic acids, Biopolymers: Original Research on Biomolecules 56 (2000) 257-265.
[15] Y. Sun, P.A. Kollman, Hydrophobic solvation of methane and nonbond parameters of the TIP3P water model, Journal of computational chemistry 16 (1995) 1164-1169.
[16] V. Zoete, M.A. Cuendet, A. Grosdidier, O. Michielin, SwissParam: a fast force field generation tool for small organic molecules, Journal of computational chemistry 32 (2011) 2359-2368.

[17] G. Binnig, C.F. Quate, C. Gerber, Atomic Force Microscope, Physical Review Letters 56 (1986) 930-933.
[18] Q.V. Vuong, T.T. Nguyen, M.S. Li, A new method for navigating optimal direction for pulling ligand from binding pocket: application to ranking binding affinity by steered molecular dynamics, J. Chem. Inf. Model. 55 (2015) 2731-2738.
